# The association of moderate-to-vigorous physical activity and sedentary behaviour with abdominal aortic calcification

**DOI:** 10.1186/s12967-023-04566-w

**Published:** 2023-10-09

**Authors:** Chang Sheng, Weihua Huang, Wei Wang, Guoqiang Lin, Mingmei Liao, Pu Yang

**Affiliations:** 1 Department of Vascular Surgery, Xiangya Hospital, Central South University, Changsha, Hunan China; 2 Department of Clinical Pharmacology, Hunan Key Laboratory of Pharmacogenetics, Xiangya Hospital, Central South University, Changsha, Hunan China; 3 Institute of Clinical Pharmacology, Hunan Key Laboratory of Pharmacogenetics Xiangya Hospital, Central South University, Changsha, Hunan China; 4 National Clinical Research Center for Geriatric Disorders, School of Pharmacy, Hunan University of Chinese Medicine, Changsha, Hunan China; 5 National Clinical Research Center for Geriatric Disorders, Xiangya Hospital, Central South University, Changsha, Hunan China; 6 Department of Cardiovascular Surgery, Xiangya Hospital, Central South University, Changsha, Hunan China; 7 Xiangya Hospital, National Health Commission Key Laboratory of Nanobiological Technology, Central South University, Changsha, Hunan China

**Keywords:** Physical activity, Sedentary behavior, Abdominal aortic calcification, Cardiovascular risk factors, NHANES

## Abstract

**Background and aims:**

The increasing prevalence of metabolic and cardiovascular diseases poses a significant challenge to global healthcare systems. Regular physical activity (PA) is recognized for its positive impact on cardiovascular risk factors. This study aimed to investigate the relationship between moderate-to-vigorous physical activity (MVPA), sedentary behavior (SB), and abdominal aortic calcification (AAC) using data from the National Health and Nutrition Examination Survey (NHANES).

**Methods:**

The study used data from NHANES participants aged 40 and above during the 2013–2014 cycle. AAC scores were assessed using the Kauppila scoring system, and MVPA and SB were self-reported. Sociodemographic variables were considered, and multivariable linear regression models were used to analyze associations between MVPA, SB, and AAC scores. Subgroup analyses were conducted based on age, sex, BMI, hypertension, and diabetes.

**Results:**

The study included 2843 participants. AAC prevalence was higher in older age groups, smokers, and those with diabetes or hypertension. Lower socioeconomic status was associated with higher AAC prevalence. Individuals engaged in any level of MVPA exhibited lower AAC rates compared to inactive individuals. Not engaging in occupational MVPA (β = 0.46, 95% confidence interval = 0.24‒0.67, *p* < .001) and prolonged SB (β = 0.28, 95% confidence interval = 0.04‒0.52, *p* = .023) were associated with higher AAC scores. However, no significant associations were found for transportation and leisure time MVPA. Subgroup analysis revealed age and hypertension as effect modifiers in the MVPA-AAC relationship.

**Conclusions:**

This study highlights the potential benefits of engaging in occupational MVPA and reducing SB in mitigating AAC scores, particularly among older individuals and those with hypertension.

**Supplementary Information:**

The online version contains supplementary material available at 10.1186/s12967-023-04566-w.

## Introduction

The changing demographics and the escalating prevalence of metabolic and cardiovascular diseases present substantial challenges to individual health and global healthcare systems. Generally, exercise is widely recognized as an effective intervention for positively influencing cardiovascular risk factors across diverse populations. Extensive evidence suggests that regular exercise impacts parameters associated with the metabolic syndrome (MetSyn), a cluster of cardiovascular risk factors [1].

A longitudinal study conducted on a population scale revealed that increased levels of moderate-to-vigorous physical activity (MVPA) and reduction in sedentary behavior (SB) were both linked to a decelerated age-related advancement of aortic stiffness, irrespective of conventional vascular risk factors [2]. Cross-sectional investigations have also demonstrated inverse connections between physical activity (PA) and diverse assessments of arterial stiffness among the elderly [3–9]. Vascular calcification (VC) refers to a pathological process involving the anomalous accumulation of minerals within the media or lipoproteins within the intima, resulting in vessel stiffening [10]. Abdominal aortic calcification (AAC), a frequent manifestation of VC [11], exhibits high prevalence in the general population, with both its incidence and severity escalating with advancing age [12]. Recently, VC has gained recognition as a protective factor that contributes to the stabilization of the aortic aneurysmal wall, likely mitigating the progression of abdominal aortic aneurysm expansion [13, 14]. However, conversely, heightened severity of aortic calcifications may lead to adverse health outcomes. It has also been found to predict cardiovascular events and all-cause mortality [15–19]. Several epidemiological studies have shown that AAC is associated with incident coronary heart disease [20], myocardial infarction [21] and stroke [22]. A more holistic risk mitigation approach, encompassing lifestyle modification or PA, may serve as a credible strategy for diminishing the deposition and advancement of VC [23]. Nonetheless, a substantial prospective study involving healthy populations revealed a counterintuitive finding: elevated levels of PA were linked to a heightened prevalence and accelerated progression of coronary artery calcium (CAC) [24]. Notably, exercise training appears ineffective in impeding the advancement of AAC among patients with chronic kidney disease [25]. Furthermore, outcomes from a study involving older men afflicted with osteosarcopenia suggest that high-intensity resistance training yielded no discernible benefits on AAC over an 18-month observation period [26]. These contradictory results suggest that the relationship of PA and SB with AAC remains unclear.

Thus, our objective was to examine the prevalence of AAC in individuals categorized as physically inactive (sedentary) relative to their physically active counterparts. Additionally, we sought to explore the correlation between MVPA and SB with AAC within the US populace, leveraging data from the National Health and Nutrition Examination Survey (NHANES). The authors postulated that elevated MVPA levels and reduced SB would correlate with a diminished AAC score.

## Methods

### Study population

We extracted data from the NHANES, a representative cross-sectional survey conducted to gather comprehensive health and nutritional information among individuals in the United States. Our study focused on NHANES participants aged 40 years or older during the 2013–2014 cycle. During this period, AAC scores were exclusively assessed within this age cohort and cycle. Our inclusion criteria encompassed individuals with complete AAC measurements, self-reported MVPA and SB data, as well as pertinent demographic variables. Notably, participants who self-reported implausible values in their PA questionnaires were meticulously excluded. Initially, our study cohort comprised 10,175 participants. After excluding individuals aged below 40 years (n = 6360), those with incomplete AAC data (n = 675), insufficient MVPA and SB data (n = 22), and those with missing covariate information (n = 275), including variables such as poverty–income ratio (PIR) (N = 250), body mass index (N = 20), education level (N = 4), marital status (N = 3), and smoking status (N = 2), a final analytic sample of 2843 participants was established (Additional file 1: Fig. S1).

Out of the 3815 participants aged 40 years and above, a cohort of 2843 individuals possessed comprehensive datasets, constituting the core analysis dataset. The cohort incorporated into the analysis exhibited prevalent attributes of advanced age, female gender, obesity, and single marital status. Moreover, a notable prevalence of diminished PIR and limited educational attainment was observed among the participants included in the study (Additional file 1: Table S1). It is imperative to acknowledge that these discernible distinctions between the included and excluded participant subsets underscore the potential limitations in extrapolating the study outcomes to broader segments of the population.

### Study variables

#### Sociodemographic characteristics

Several key sociodemographic variables were meticulously evaluated, including age, gender (male/female), race-ethnicity (categorized as Mexican American, other Hispanic, non-Hispanic White, non-Hispanic Black, non-Hispanic Asian, and others), Body Mass Index (BMI), marital status (classified as married/living with partner or single), educational attainment (stratified into high school degree/equivalency or less, some college or associates degree, college graduate or above), and PIR, smoking status (classified as non-smoker, former smoker or current smoker), diabetes (yes/no) and hypertension (yes/no) [27–29]. We categorized BMI as normal (kg/m²<25.0), overweight (25.0 ≤ kg/m²<30.0), or obese (kg/m²≥30.0) [30, 31]. The marital status and educational attainment of participants adhered to definitions established in prior research [32]. The PIR quantified income in relation to federal poverty thresholds, with a range of 0 (no income) to 5 (greater than or equal to five times the federal poverty level) [33]. Smoking status was categorized based on reported cigarettes smoked: participants reporting fewer than 100 cigarettes in their lifetime were designated as “never smokers”, those reporting ≥ 100 cigarettes and currently smoking “every day” or “some days” were classified as “current smokers” and individuals who reported ≥ 100 cigarettes but had quit smoking at the time of the questionnaire were identified as “former smokers” [34]. Hypertension diagnosis criteria encompassed average systolic blood pressure (ASBP) / the average diastolic blood pressure (ADBP) ≥ 140/90 mmHg, antihypertensive medication usage, and history of hypertension [32]. The calculation methods of ASBP and ADBP have been reported in previous study [16]. Participants were classified as having diabetes if they met any of the following criteria: (1) a random glucose level equal to or exceeding 11.1 mmol/L, (2) a fasting glucose level exceeding 7 mmol/L, (3) a glycated hemoglobin A1c level equal to or surpassing 6.5%, (4) a blood glucose level of ≥ 11.1 mmol/L during a 2 h oral glucose tolerance test (OGTT), (5) ongoing treatment with hypoglycemic medications, or (6) a previous medical diagnosis of diabetes. [32].

#### Primary outcome: AAC

The severity of AAC was assessed using the Kauppila scoring system, which entailed analysis of lateral spine images obtained via dual-energy X-ray absorptiometry (DXA). Participants younger than 40 years, pregnant individuals, recent users of radiographic contrast, those exceeding 450 pounds in weight, or those with Harrington rod-related scoliosis were excluded from DXA examination. The AAC-24 scoring methodology divided the anterior and posterior aortic walls into segments corresponding to lumbar spine regions L1–L4. AAC severity was quantified using a 0–3 score for each of the eight segments, indicative of the degree of calcification. The total AAC score ranged from 0 to 24, with higher scores reflecting more pronounced calcification. Notably, an AAC score above 6 was employed as a widely recognized threshold for severe AAC (SAAC) [32, 35].

#### Primary exposure: self-reported MVPA and SB

Data on self-reported MVPA and SB were derived from the Global Physical Activity Questionnaire [36]. MVPA is defined as activities that lead to a significant increase in respiration or heart rate. This questionnaire encompassed various domains of MVPA, including transportation, occupational, and leisure-time MVPA, as well as sedentary time domains. The total MVPA duration was calculated by aggregating the reported time across each domain. We classified MVPA and its constituent domains into binary categories: “none” denoted MVPA of 0 min/week, while “any” encompassed MVPA exceeding 0 min/week. [33]. These categorizations were chosen due to the notable skewness in the data, with approximately 27% of participants reporting zero MVPA minutes. For instance, 77% reported no transportation MVPA, 65% reported no occupational MVPA, and 51% reported no leisure-time MVPA. Similarly, close to 50% reported high daily hours of SB. Total sedentary behavior time (TSBT) was classified as either high (reporting more than 7.5 h/day) or low (reporting 7.5 h/day or less), following prior definitions provided by NHANES participants [33].

### Statistical methods

In accordance with Center for Disease Control and Prevention (CDC) guidelines, all statistical analyses were conducted using appropriate NHANES sampling weights to address the complexities inherent in the multi-stage cluster survey design. This approach ensured accurate representation of the broader population [37]. Participant characteristics, with respect to AAC, were presented as percentages for categorical variables. We presented descriptive statistics detailing the prevalence of AAC across MVPA, TSBT, and individual MVPA domains. We employed the Wilcoxon rank sum test and chi-square test to assess clinical characteristic differences between included and excluded populations. To gauge the sociodemographic disparities across AAC, we employed Pearson’s chi-squared test or Fisher’s exact test for categorical variables. Associations linking MVPA, SB, and AAC scores were evaluated through weighted univariable and multivariable linear regression models. Model 1 remained unadjusted, while Model 2 incorporated adjustments for age, sex, and race. Model 3 further accounted for additional covariates, including age, sex, race, BMI, marital status, education level, PIR, smoking, hypertension, and diabetes. To explore potential non-linear relationships, the restricted cubic spline (RCS) was applied to examine TSBT’s influence on AAC score. Additionally, we conducted subgroup analyses through stratified multivariable linear regression models, factoring in variables such as sex, age, BMI, hypertension, and diabetes as potential effect modifiers. To quantify heterogeneity, interaction terms were introduced and evaluated using the likelihood ratio test. These analyses were conducted employing survey methods in R (version 4.2.1) software to account for the intricate survey design’s stratification, clustering, and weighting of applying Mobile Examination Center (MEC) weights. Statistical significance was established at a two-tailed *p* < .05.

## Results

### Participants characteristic

Various sociodemographic groups exhibited statistically significant higher rates of AAC and SAAC (Table 1). Advanced age, non-Hispanic White ethnicity, single marital status, and smoking were associated with elevated AAC rates. Conversely, a lower prevalence of AAC was observed in obese participants. Notably, individuals with diabetes, hypertension, lower PIR, or limited educational attainment demonstrated significantly higher AAC prevalence compared to their counterparts. Similar trends were evident in SAAC rates, except for BMI status, education level, and PIR (all *p* > .05)..

**Table 1:** Demographics and comparisons between AAC status and severity among covariates

Characteristic	All (n = 2843) (%)	AAC					
		No (n = 1981) (% of All)	Yes			*p*-Value Yes/No	*p*-Value Severe/non-Severe
			All yes (n = 862) (% of all)	non-SAAC (n = 601) (% of all Yes)	SAAC (n = 261) (% of all Yes)		
Age (years)						< 0.001***	< 0.001***
40–59	1526 (53.68)	1237 (81.06)	289 (18.94)	257 (88.93)	32 (11.07)		
60+	1317 (46.32)	744 (56.49)	573 (43.51)	344 (60.03)	229 (39.97)		
Sex (%)						0.308	0.286
Male	1377 (48.43)	947 (68.77)	430 (31.23)	307 (71.40)	123 (28.60)		
Female	1466 (51.57)	1034 (70.53)	432 (29.47)	294 (68.06)	138 (31.94)		
Race (%)						< 0.001***	< 0.001***
Mexican American	347 (12.21)	264 (76.08)	83 (23.92)	59 (71.08)	24 (28.92)		
Other Hispanic	256 (9.00)	191 (74.61)	65 (25.39)	54 (83.08)	11 (16.92)		
Non-Hispanic White	1286 (45.23)	812 (63.14)	474 (36.86)	303 (63.92)	171 (36.08)		
Non-Hispanic Black	567 (19.94)	425 (74.96)	142 (25.04)	107 (75.35)	35 (24.65)		
Non-Hispanic Asian	330 (11.61)	248 (75.15)	82 (24.85)	65 (79.27)	17 (20.73)		
Other Race	57 (2.00)	41 (71.93)	16 (28.07)	13 (81.25)	3 (18.75)		
BMI status						< 0.001***	0.112
Normal (< 25 kg/m^2^)	806 (28.35)	548 (67.99)	258 (32.01)	171 (66.28)	87 (33.72)		
Overweight (≥ 25 and < 30 kg/m^2^)	1033 (36.33)	677 (65.54)	356 (34.46)	245 (68.82)	111 (31.18)		
Obese (≥ 30 kg/m^2^)	1004 (35.31)	756 (75.30)	248 (24.70)	185 (74.60)	63 (25.40)		
Marital status (%)						< 0.001***	< 0.001***
Married/Living with partner	1799 (63.28)	1304 (72.48)	495 (27.52)	369 (74.55)	126 (25.45)		
Single	1044 (36.72)	677 (64.85)	367 (35.15)	232 (63.22)	135 (36.78)		
Education level (%)						0.034*	0.346
High school degree/equivalency or less	1258 (44.25)	851 (67.65)	407 (32.35)	274 (67.32)	133 (32.68)		
Some college or associates degree	822 (28.91)	572 (69.59)	250 (30.41)	179 (71.60)	71 (28.40)		
College Graduate or above	763 (26.84)	558 (73.13)	205 (26.87)	148 (72.20)	57 (27.80)		
PIR						0.002**	0.535
<1.38	886 (31.16)	609 (68.74)	277 (31.26)	190 (68.59)	87 (31.41)		
≥1.38 and < 3.99	1129 (39.71)	757 (67.05)	372 (32.95)	256 (68.82)	116 (31.18)		
≥3.99	828 (29.12)	615 (74.28)	213 (25.72)	155 (72.77)	58 (27.2)		
Smoking (%)						< 0.001***	0.014*
Never	1503 (52.87)	1112 (73.99)	391 (26.01)	290 (74.17)	101 (25.83)		
Former	803 (28.24)	513 (63.89)	290 (36.11)	185 (63.79)	105 (36.21)		
Now	537 (18.89)	356 (66.29)	181 (33.71)	126 (69.61)	55 (30.39)		
Diabetes (%)						< 0.001***	< 0.001***
Yes	668 (23.50)	400 (59.88)	268 (40.12)	165 (61.57)	103 (38.43)		
No	2175 (76.50)	1581 (72.69)	594 (27.31)	436 (73.40)	158 (26.60)		
Hypertension (%)						< 0.001***	< 0.001***
Yes	1367 (48.08)	839 (61.38)	528 (38.62)	332 (62.88)	196 (37.12)		
No	1476 (51.92)	1142 (77.85)	334 (22.15)	269 (80.54)	65 (19.46)		

Data is presented as proportions for categorical variables, n (%)

*p*-Value indicates varying proportions of AAC or AAC severity based on sex, age, race, BMI status, marital status, education, poverty level, smoking status, diabetes, and hypertension

Analysis conducted: Chi-square test

* AAC* abdominal aortic calcification; *SAAC* severe abdominal aortic calcification; *BMI* body mass index; *PIR* poverty income ratio

*p < .05;

**p < .01;

***p < .001

### AAC by MVPA and SB

The findings in Table 2 reveal that individuals engaging in any level of MVPA (greater than 0 min/week) exhibited lower AAC rates (27.62% vs. 37.66%, *p* < .001) compared to those with no MVPA (0 min/week). Additionally, individuals with lower TSBT demonstrated decreased AAC rates relative to those with higher TSBT (28.39% vs. 32.39%, *p* = .020). Among the three MVPA domains, any time allocated to MVPA was associated with a reduced AAC prevalence compared to individuals reporting no MVPA (transportation: 27.15% vs. 31.26%, *p* = .045; occupational: 26.74% vs. 32.28%, *p* = .002; leisure: 26.74% vs. 33.77%, *p* < .001).

**Table 2:** AAC status and severity by level of physical activity and sedentary behaviour

Characteristic	All (n = 2843) (%)	AAC					
		No (n = 1981) (% of All)	Yes			*p*-Value Yes/No	*p*-Value Severe/non-Severe
			All yes (n = 862) (% of all)	non-Severe AAC (n = 601) (% of all Yes)	Severe AAC (n = 261) (% of all Yes)		
MVPA						< 0.001***	0.039*
Any (> 0 min/week)	2081 (73.20)	1506 (72.38)	575 (27.62)	414 (72.00)	161 (28.00)		
None (0 min/week)	762 (26.80)	475 (62.34)	287 (37.66)	187 (65.16)	100 (34.84)		
Transportation MVPA						0.045*	0.013*
Any (> 0 min/week)	652 (22.93)	475 (72.85)	177 (27.15)	137 (77.40)	40 (22.60)		
None (0 min/week)	2191 (77.07)	1506 (68.74)	685 (31.26)	464 (67.74)	221 (32.26)		
Occupational MVPA						0.002**	0.021*
Any (> 0 min/week)	1006 (35.39)	737 (73.26)	269 (26.74)	202 (75.09)	67 (24.91)		
None (0 min/week)	1837 (64.61)	1244 (67.72)	593 (32.28)	399 (67.28)	194 (32.72)		
Leisure MVPA						< 0.001***	0.874
Any (> 0 min/week)	1395 (49.07)	1022 (73.26)	373 (26.74)	259 (69.44)	114 (30.56)		
None (0 min/week)	1448 (50.93)	959 (66.23)	489 (33.77)	342 (69.94)	147 (30.06)		
TSBT						0.020*	0.353
Low ≤ 7.5 h/day	1469 (51.67)	1052 (71.61)	417 (28.39)	297 (71.22)	120 (28.78)		
High > 7.5 h/day	1374 (48.33)	929 (67.61)	445 (32.39)	304 (68.31)	141 (31.69)		

Data is presented as proportions for categorical variables, n (%)

*p*-Value indicates varying proportions of AAC or AAC severity based on MVPA, transportation MVPA, occupational MVPA, leisure-time MVPA, and TSBT.

Analysis conducted: Chi-square test

*AAC* abdominal aortic calcification; *SAAC* severe abdominal aortic calcification; *MVPA* moderate-to-vigorous physical activity; *TSBT* total sedentary behaviour time

*p < .05;

**p < .01;

***p < .001

Moreover, among individuals with AAC, no significant disparity in SAAC prevalence was observed between those engaged in any time versus no time leisure MVPA (30.56% vs. 30.06%, *p* = .874). Additionally, there was no substantial divergence in SAAC prevalence between low and high TSBT groups (28.78% vs. 31.69%, *p* = .353) (Table 2).

### MVPA and SB is associated with increased AAC score

**Fig. 1:**
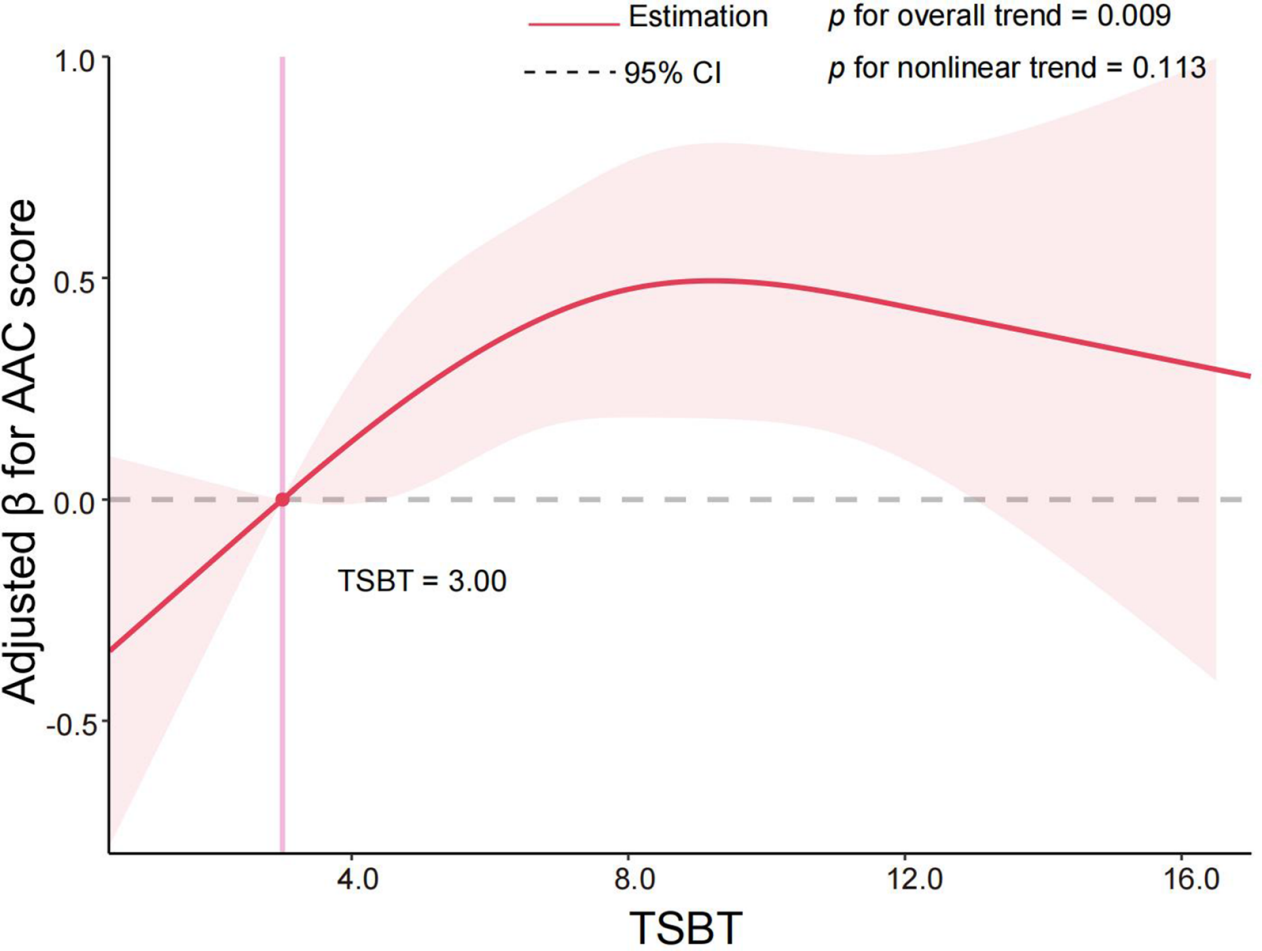
Restricted spline curve shows the relationship between TSBT and AAC scores. Red line and red transparent area represent beta and 95% CI, respectively; the unit of TSBT is hours. β (95% CI) was adjusted based on Model 3 (sex, age, race, BMI, marital status, education, poverty level, smoking status, diabetes, and hypertension). *AAC* abdominal aortic calcification; *TSBT* total sedentary behaviour time; *CI* confidence interval

Results from our adjusted multivariable linear regression model (Table 3) reveal associations between MVPA and occupational MVPA with AAC score. Specifically, individuals reporting no MVPA or occupational MVPA exhibited higher AAC scores (β = 0.40, 95% confidence interval [CI]  0.09‒0.71, *p* = .013; β = 0.46, 95% CI  0.24‒0.67, *p* < .001). In unadjusted models, participants reporting no leisure time MVPA demonstrated higher AAC scores (β = 0.39, 95% CI  0.04‒0.75, *p* = .028); however, this association lost significance upon covariate adjustment. Similar trends were noted for no transportation MVPA, with higher AAC scores observed in the minimally adjusted model (β = 0.29, 95% CI   0.03‒0.55, *p* = .026). While high TSBT was not associated with higher AAC scores in crude and minimally adjusted models, a significant association emerged upon full covariate adjustment (β = 0.28, 95% CI   0.04‒0.52, *p* = .023). Moreover, multivariable-adjusted RCS analysis demonstrated a notable dose-response relationship between TSBT and AAC score (*p* for overall trend = 0.009; *p* for nonlinear trend = 0.113) (Fig. 1). The curve exhibited a negative β when TSBT < 3.00, followed by a rising trajectory until TSBT reached approximately nine. Subsequently, a gradual decline in β was observed. Notably, extreme TSBT values at both ends did not yield statistically significant correlations with AAC scores.

**Table 3:** Multivariable linear regression to determine the β (95% CIs) of AAC scores presence by physical activity or sedentary behaviour

	Crude model (Model 1)	*p*-value	Minimally adjusted model (Model 2)	*p*-value	Fully adjusted model (Model 3)	*p*-value
AAC Score/β (95% CI)						
MVPA						
None (0 min/week)	0.75 (0.48, 1.03)	< 0.001***	0.49 (0.22, 0.76)	< 0.001***	0.40 (0.09, 0.71)	0.013*
Any (> 0 min/week)	Reference		Reference		Reference	
Transportation MVPA						
None (0 min/week)	0.44 (0.20, 0.68)	< 0.001***	0.29 (0.03, 0.55)	0.026*	0.28 (0.00, 0.57)	0.053
Any (> 0 min/week)	Reference		Reference		Reference	
Occupational MVPA						
None (0 min/week)	0.45 (0.16, 0.73)	0.002**	0.36 (0.14, 0.58)	0.001**	0.46 (0.24, 0.67)	< 0.001***
Any (> 0 min/week)	Reference		Reference		Reference	
Leisure time MVPA						
None (0 min/week)	0.39 (0.04, 0.75)	0.028*	0.24 (− 0.14, 0.62)	0.449	0.04 (− 0.43, 0.51)	0.868
Any (> 0 min/week)	Reference		Reference		Reference	
TSBT						
Hight > 7.5 h/day	0.15 (− 0.08, 0.38)	0.196	0.20 (− 0.04, 0.44)	0.097	0.28 (0.04, 0.52)	0.023*
Low ≤ 7.5 h/day	Reference		Reference		Reference	

Data are presented as β, 95% CIs (confidence intervals), and *p*-value; physical activity domains categorized in either any or none; omitted for collinearity

Model 1 was adjusted for none

Model 2 was adjusted for age, sex, race

Model 3 was adjusted for sex, age, race, BMI, marital status, education, poverty level, smoking status, diabetes, and hypertension

*AAC* abdominal aortic calcification; *MVPA* moderate-to-vigorous physical activity; *TSBT* total sedentary behaviour time

*p < .05;

**p < .01;

***p < .001

### Subgroup analysis

**Fig. 2:**
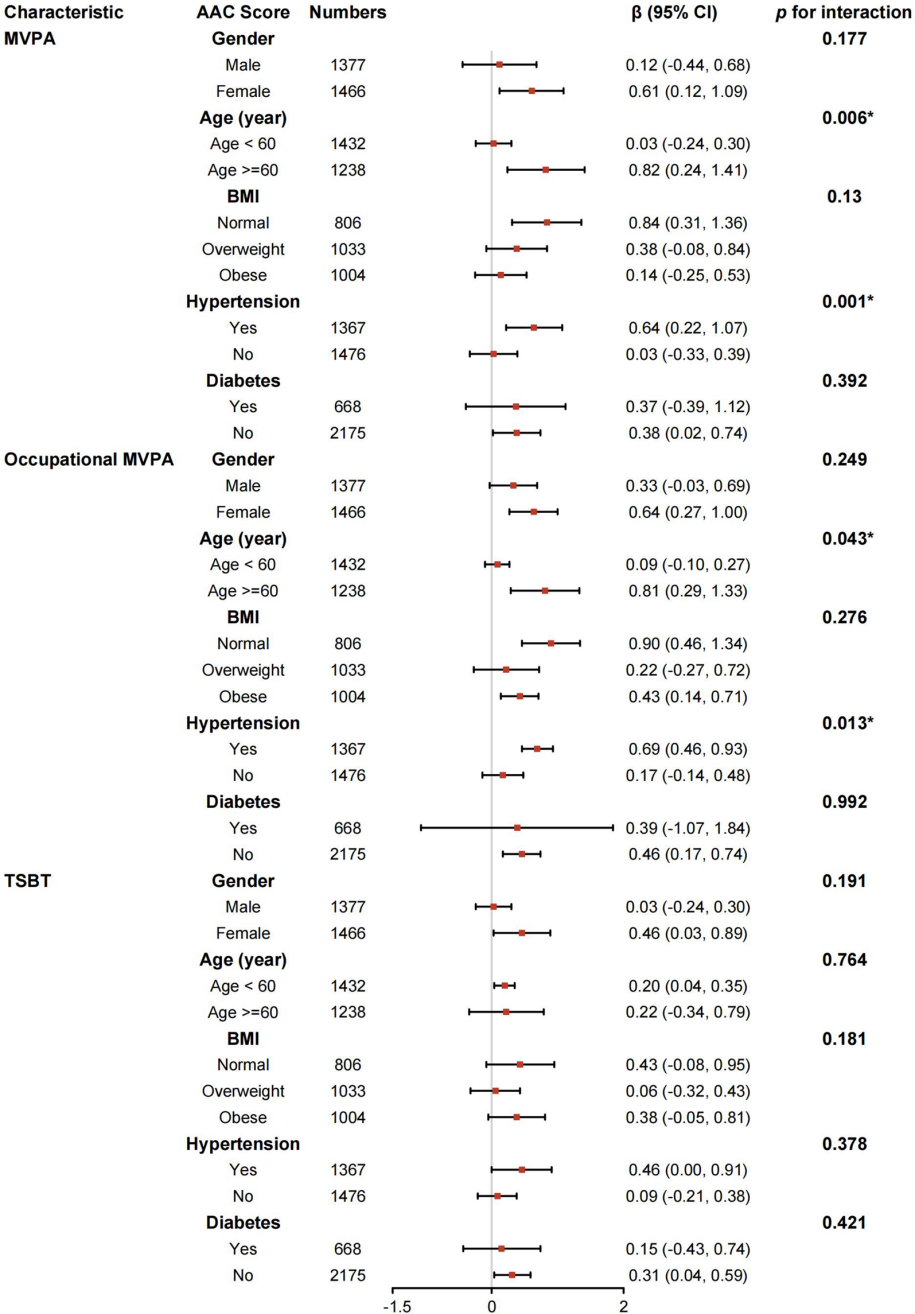
Subgroup analysis for the association between MVPA, Occupational MVPA or TSBT and AAC. The results were adjusted for all covariates except the corresponding stratification variable. *AAC* abdominal aortic calcification; *BMI* body mass index; *MVPA* moderate-to-vigorous physical activity; *TSBT* total sedentary behaviour time. *p < .05

To assess the consistency of associations between MVPA, occupational MVPA, or TSBT with AAC score across diverse populations, we conducted subgroup analyses and interaction tests stratified by age, sex, BMI, hypertension, and diabetes. Our findings regarding MVPA demonstrated heterogeneous associations. Figure 2 reveals significant interactions related to age and hypertension (all *p* for interaction < 0.05), while no substantial interactions were detected for sex, BMI, or diabetes. MVPA remained positively associated with AAC score among females, those aged ≥ 60, individuals with normal BMI, hypertension, and those without diabetes. In sum, our results underscore the dependence of the MVPA–AAC score association on age subgroups and hypertension status, it may be appropriate for those age ≥ 60 or with hypertension.

Similarly, for occupational MVPA, interactions were detected for age (*p* for interaction = 0.043) and hypertension (*p* for interaction = 0.013). Notably, a positive association was discerned among females, individuals aged ≥ 60, those with normal BMI, obesity, hypertension, and non-diabetic participants. Conversely, interactions for sex, BMI, and diabetes demonstrated no significant differences (all *p* for interaction > 0.05). Overall, our findings underscore the dependence of the occupational MVPA–AAC score association on age and hypertension status, especially among individuals aged ≥ 60 or with hypertension (Fig. 2).

Furthermore, interaction tests indicated no significant differences in the association between TSBT and AAC score across various stratifications. These results suggest a lack of substantial dependence on gender, age, BMI, hypertension, and diabetes for this positive association (all *p* for interaction > 0.05) (Fig. 2).

## Discussion

### Principal findings

Within our study population, AAC was prevalent in approximately 30% of individuals. Notably, a higher prevalence of AAC was observed among individuals aged 60 years or older, those identifying as non-Hispanic White, those who were single, smokers, and those with comorbidities such as diabetes and hypertension. Additionally, lower levels of PIR and educational attainment were associated with higher AAC prevalence. Conversely, a lower prevalence of AAC was found among obese participants. Furthermore, our analysis revealed that AAC occurrence was more common in individuals engaged in lower levels of MVPA and those with higher levels of SB. After adjusting for various confounding factors, we established that being physically active (engaging in occupational MVPA or maintaining SB of ≤ 7.5 h per day) held the potential to mitigate AAC scores. Notably, the other two types of MVPA showed no significant association with AAC scores. These findings underscore the positive impact of engaging in occupational MVPA and reducing SB on AAC scores.

### Comparisons with other studies

Our observations align with prior research indicating that smoking, disadvantaged socioeconomic backgrounds, and lower educational levels correlate with a higher likelihood of AAC [27, 28, 38, 39]. Furthermore, the finding that AAC prevalence is higher among non-Hispanic Whites is consistent with the Multi-Ethnic Study of Atherosclerosis (MESA) [39]. Intriguingly, our study uncovered a negative correlation between increased BMI and AAC occurrence, suggesting a potential protective role of higher BMI. This concurs with previous studies demonstrating a similar inverse relationship [38, 40, 41].

The well-established association between physical inactivity, prolonged SB, and elevated cardiovascular disease risk is reaffirmed in our study [42, 43]. Our findings align with an earlier small retrospective study that reported a negative correlation between exercise and AAC [28]. However, among the three distinct types of MVPA examined, a significant negative correlation with AAC scores was only evident for occupational MVPA, even after accounting for other influencing factors. Currently, knowledge in this field is scarce. Previous research has indicated an association between occupational PA and a heightened risk of cardiovascular diseases [44–46]. A plausible explanation for the occupational PA health paradox lies in inadequate recovery time during and after such activities, contributing to an increased 24 h cardiovascular load [47]. Future studies might explore the dose-response relationship between heightened occupational MVPA and AAC mitigation, despite mixed outcomes in prior research [26]. Our findings regarding occupational MVPA contribute to the understanding of this health paradox phenomenon. While research has indicated that as daily SB extends, there is an increase in the prevalence of coronary artery calcification [48, 49], our results lack direct comparability with existing studies concerning the relationship between SB and AAC. Nevertheless, our findings align with the perspective that reducing SB yields cardiovascular benefits [50].

Subgroup analysis has provided invaluable insights into potential interactions between MVPA, age, hypertension, and their collective influence on AAC scores. Particularly in individuals of advanced age or those with hypertension, the association between MVPA and AAC scores becomes more pronounced, highlighting the heightened significance of the MVPA-AAC connection in these contexts.

### Potential mechanisms

The benefits of PA and reduced SB extend beyond metabolic and cardiovascular improvements to encompass musculoskeletal health, especially pertinent to older individuals [51, 52]. Wu et al. demonstrated that participants with osteoporosis had higher AAC scores, and osteoporosis was positively associated with SAAC independent on demographics and other factors relevant to AAC [53]. However, the effects of exercise on diverse outcomes are contingent on exercise type and strain composition.

While the precise biological mechanisms linking PA to AAC remain inadequately understood, existing knowledge connects physical exercise to improved vascular compliance and remodeling, enhanced endothelial function through increased laminar shear stress, reduced production of vasoconstrictors, oxidative stress, and low-grade inflammation [54–61]. These mechanisms collectively support the protective role of PA, particularly sports activities, against aortic stiffness. Oxidative stress in vascular smooth muscle cells triggers signals like Wnt, leading to osteogenic differentiation in vascular walls, possibly contributing to calcium deposition and inflammation, fostering the calcification cycle [62, 63]. It is acknowledged that the intricate pathogenesis of AAC development goes beyond this paper’s scope.

### Strengths, limitations and future directions

Our analysis boasts several strengths. The categorization of MVPA enables a nuanced exploration of its AAC association, facilitating comparisons with proposed mechanisms. Additionally, the study’s population-based, nationally representative survey design enhances the generalizability of findings to the broader US population. Moreover, novel stratified analyses address confounding factors and inconsistencies surrounding the occupational MVPA health paradox.

Notwithstanding these strengths, limitations exist. The reliance on self-reported MVPA and SB is acknowledged. Patients with AAC are more likely to have comorbidities that lead to reduced PA or prolonged SB. The cross-sectional design raises questions of causality, necessitating further experimental research to establish causal relationships and elucidate mechanisms. While the study assumes temporality in a short timeframe, systematic changes in these measures are unlikely. Our study underscores the significance of MVPA and suggests that interventions to enhance MVPA and reduce SB could yield substantial effects on AAC. Further investigation is warranted to unravel the synergistic effects of different forms of PA within the complex pathophysiology of AAC. Future research also should employ improved methodologies to validate these findings, including longitudinal designs and enhanced measurements of MVPA and SB.

## Conclusions

To conclude, our study underscores the potential of increasing PA levels and minimizing SB to confer favorable vascular effects and decelerate the progression of AAC. This observation offers a plausible explanation for the intricate connection between physical inactivity and the elevated risk of cardiovascular diseases. As we navigate the landscape of preventive cardiovascular health, these findings underscore the significance of lifestyle interventions centered around promoting PA and curtailing sedentary habits.

### Supplementary Information

**Additional file 1: Figure S1.** Flowchart of the sample selection from NHANES 2013–2014. Missing covariate information (n = 275), including variables such as lower poverty–income ratio (PIR) (N = 250), body mass index (N = 20), education level (N = 4), marital status (N = 3), and smoking status (N = 2). *NHANES* National Health and Nutrition Examination Survey; *AAC* abdominal aortic calcification; *MVPA* moderate-to-vigorous physical activity; *SB* sedentary behavior. **Table S1.** Baseline characteristics of the included and excluded populations.

## Data Availability

Publicly available datasets were analyzed in this study. This data can be found here: https://wwwn.cdc.gov/nchs/nhanes/ (Accessed on 1 Aug 2023).

## References

[1] Alberti KG, Zimmet P, Shaw J (2005). The metabolic syndrome–a new worldwide definition. Lancet.

[2] Ahmadi-Abhari S, Sabia S, Shipley MJ, Kivimäki M, Singh-Manoux A, Tabak A, McEniery C, Wilkinson IB, Brunner EJ (2017). Physical activity, sedentary behavior, and long-term changes in aortic stiffness: the Whitehall II study. J Am Heart Assoc.

[3] Karimi L, Mattace-Raso FU, van Rosmalen J, van Rooij F, Hofman A, Franco OH (2016). Effects of combined healthy lifestyle factors on functional vascular aging: the Rotterdam Study. J Hypertens.

[4] Endes S, Schaffner E, Caviezel S, Dratva J, Autenrieth CS, Wanner M, Martin B, Stolz D, Pons M, Turk A (2016). Physical activity is associated with lower arterial stiffness in older adults: results of the SAPALDIA 3 cohort study. Eur J Epidemiol.

[5] Endes S, Schaffner E, Caviezel S, Dratva J, Autenrieth CS, Wanner M, Martin B, Stolz D, Pons M, Turk A (2016). Long-term physical activity is associated with reduced arterial stiffness in older adults: longitudinal results of the SAPALDIA cohort study. Age Ageing.

[6] Funck KL, Laugesen E, Høyem P, Fleischer J, Cichosz SL, Christiansen JS, Hansen TK, Poulsen PL (2016). Low physical activity is associated with increased arterial stiffness in patients recently diagnosed with type 2 diabetes. Am J Hypertens.

[7] Parsons TJ, Sartini C, Ellins EA, Halcox JPJ, Smith KE, Ash S, Lennon LT, Wannamethee SG, Lee IM, Whincup PH, Jefferis BJ (2016). Objectively measured physical activity, sedentary time and subclinical vascular disease: cross-sectional study in older british men. Prev Med.

[8] Laursen ASD, Hansen AS, Wiinberg N, Brage S, Sandbæk A, Lauritzen T, Witte DR, Jørgensen ME, Johansen NB (2015). Higher physical activity is associated with lower aortic stiffness but not with central blood pressure: the ADDITION-pro study. Med (Baltim).

[9] Crichton GE, Elias MF, Robbins MA (2014). Cardiovascular health and arterial stiffness: the Maine-Syracuse longitudinal study. J Hum Hypertens.

[10] Quaglino D, Boraldi F, Lofaro FD (2020). The biology of vascular calcification. Int Rev Cell Mol Biol.

[11] Reaven PD, Sacks J (2004). Reduced coronary artery and abdominal aortic calcification in Hispanics with type 2 diabetes. Diabetes Care.

[12] Bartstra JW, Mali W, Spiering W, de Jong PA (2021). Abdominal aortic calcification: from ancient friend to modern foe. Eur J Prev Cardiol.

[13] Klopf J, Fuchs L, Schernthaner R, Domenig CM, Gollackner B, Brostjan C, Neumayer C, Eilenberg W (2022). The prognostic impact of vascular calcification on abdominal aortic aneurysm progression. J Vasc Surg.

[14] Nakayama A, Morita H, Hayashi N, Nomura Y, Hoshina K, Shigematsu K, Ohtsu H, Miyata T, Komuro I (2016). Inverse correlation between calcium accumulation and the expansion rate of abdominal aortic aneurysms. Circ J.

[15] Harbaugh CM, Terjimanian MN, Lee JS, Alawieh AZ, Kowalsky DB, Tishberg LM, Krell RW, Holcombe SA, Wang SC, Campbell DA, Englesbe MJ (2013). Abdominal aortic calcification and surgical outcomes in patients with no known cardiovascular risk factors. Ann Surg.

[16] Echouffo-Tcheugui JB, Allison M, Kalyani RR, Sims M, Bertoni AG, Golden SH (2017). Abdominal aortic calcification among individuals with and without diabetes: the Jackson heart study. Diabetes Care.

[17] Lewis JR, Schousboe JT, Lim WH, Wong G, Wilson KE, Zhu K, Thompson PL, Kiel DP, Prince RL (2018). Long-term atherosclerotic vascular disease risk and prognosis in elderly women with abdominal aortic calcification on lateral spine images captured during bone density testing: a prospective study. J Bone Miner Res.

[18] Leow K, Szulc P, Schousboe JT, Kiel DP, Teixeira-Pinto A, Shaikh H, Sawang M, Sim M, Bondonno N, Hodgson JM (2021). Prognostic value of abdominal aortic calcification: a systematic review and meta-analysis of observational studies. J Am Heart Assoc.

[19] Ishii D, Sakamoto S, Okazaki T, Kuwabara M, Hosogai M, Horie N (2022). Abdominal aortic calcification volume is associated with wall enhancement of unruptured intracranial aneurysm. World Neurosurg.

[20] Chen HC, Wang WT, Hsi CN, Chou CY, Lin HJ, Huang CC, Chang CT (2018). Abdominal aortic calcification score can predict future coronary artery disease in hemodialysis patients: a 5-year prospective cohort study. BMC Nephrol.

[21] van der Meer IM, Bots ML, Hofman A, del Sol AI, van der Kuip DA, Witteman JC (2004). Predictive value of noninvasive measures of atherosclerosis for incident myocardial infarction: the Rotterdam study. Circulation.

[22] Levitzky YS, Cupples LA, Murabito JM, Kannel WB, Kiel DP, Wilson PW, Wolf PA, O’Donnell CJ (2008). Prediction of intermittent claudication, ischemic stroke, and other cardiovascular disease by detection of abdominal aortic calcific deposits by plain lumbar radiographs. Am J Cardiol.

[23] Basile C, Lomonte C, Lisi P, Karohl C, Di Iorio B, Bellasi A (2014). Physical activity in chronic kidney disease: a plausible approach to vascular calcification?. Kidney Blood Press Res.

[24] Sung KC, Hong YS, Lee JY, Lee SJ, Chang Y, Ryu S, Zhao D, Cho J, Guallar E, Lima JAC (2021). Physical activity and the progression of coronary artery calcification. Heart.

[25] Zhou Y, Hellberg M, Hellmark T, Höglund P, Clyne N (2020). Twelve months of exercise training did not halt abdominal aortic calcification in patients with CKD - a sub-study of RENEXC-a randomized controlled trial. BMC Nephrol.

[26] Knauer K, Chaudry O, Uder M, Kohl M, Kemmler W, Bickelhaupt S, Engelke K (2023). Effects of high-intensity resistance training on visceral adipose tissue and abdominal aortic calcifications in older men with Osteosarcopenia—results from the FrOST study. Clin Interv Aging.

[27] Forbang NI, McClelland RL, Remigio-Baker RA, Allison MA, Sandfort V, Michos ED, Thomas I, Rifkin DE, Criqui MH (2016). Associations of cardiovascular disease risk factors with abdominal aortic calcium volume and density: the multi-ethnic study of atherosclerosis (MESA). Atherosclerosis.

[28] Kim ED, Kim JS, Kim SS, Jung JG, Yun SJ, Kim JY, Ryu JS (2013). Association of abdominal aortic calcification with lifestyle and risk factors of cardiovascular disease. Korean J Fam Med.

[29] Onuma OK, Pencina K, Qazi S, Massaro JM, D’Agostino RB, Chuang ML, Fox CS, Hoffmann U, O’Donnell CJ (2017). Relation of risk factors and abdominal aortic calcium to progression of coronary artery calcium (from the Framingham heart study). Am J Cardiol.

[30] Tobias DK, Luttmann-Gibson H, Mora S, Danik J, Bubes V, Copeland T, LeBoff MS, Cook NR, Lee IM, Buring JE, Manson JE (2023). Association of body weight with response to vitamin D supplementation and metabolism. JAMA Netw Open.

[31] Huang Y, Xu P, Fu X, Ren Z, Cheng J, Lin Z, Tan J, Huang B, Huang Z, Xu H (2021). The effect of triglycerides in the associations between physical activity, sedentary behavior and depression: an interaction and mediation analysis. J Affect Disord.

[32] Fan H, Xiong Y, Huang Y, Li W, Xu C, Feng X, Hua R, Yang Y, Wang Z, Yuan Z, Zhou J (2023). Coffee consumption and abdominal aortic calcification among adults with and without hypertension, diabetes, and cardiovascular diseases. Nutr Metab Cardiovasc Dis.

[33] Almohamad M, Krall Kaye E, Mofleh D, Spartano NL (2022). The association of sedentary behaviour and physical activity with periodontal disease in NHANES 2011–2012. J Clin Periodontol.

[34] Tomar SL, Asma S (2000). Smoking-attributable periodontitis in the United States: findings from NHANES III. J Periodontol.

[35] Chen W, Eisenberg R, Mowrey WB, Wylie-Rosett J, Abramowitz MK, Bushinsky DA, Melamed ML (2020). Association between dietary zinc intake and abdominal aortic calcification in US adults. Nephrol Dial Transplant.

[36] Schuna JM, Johnson WD, Tudor-Locke C (2013). Adult self-reported and objectively monitored physical activity and sedentary behavior: NHANES 2005–2006. Int J Behav Nutr Phys Act.

[37] Johnson CL, Paulose-Ram R, Ogden CL, Carroll MD, Kruszon-Moran D, Dohrmann SM, Curtin LR (2013). National health and nutrition examination survey: analytic guidelines, 1999–2010. Vital Health Stat.

[38] Rahman EU, Chobufo MD, Farah F, Elhamdani A, Khan A, Thompson EA, Aronow WS, El-Hamdani M (2021). Prevalence and risk factors for the development of abdominal aortic calcification among the US population: NHANES study. Arch Med Sci Atheroscler Dis.

[39] Allison MA, Budoff MJ, Nasir K, Wong ND, Detrano R, Kronmal R, Takasu J, Criqui MH (2009). Ethnic-specific risks for atherosclerotic calcification of the thoracic and abdominal aorta (from the multi-ethnic study of atherosclerosis). Am J Cardiol.

[40] Fox CS, Hwang SJ, Massaro JM, Lieb K, Vasan RS, O’Donnell CJ, Hoffmann U (2009). Relation of subcutaneous and visceral adipose tissue to coronary and abdominal aortic calcium (from the Framingham heart study). Am J Cardiol.

[41] Tankó LB, Bagger YZ, Alexandersen P, Larsen PJ, Christiansen C (2003). Central and peripheral fat mass have contrasting effect on the progression of aortic calcification in postmenopausal women. Eur Heart J.

[42] Ding D, Lawson KD, Kolbe-Alexander TL, Finkelstein EA, Katzmarzyk PT, van Mechelen W, Pratt M (2016). The economic burden of physical inactivity: a global analysis of major non-communicable diseases. Lancet.

[43] Young DR, Hivert MF, Alhassan S, Camhi SM, Ferguson JF, Katzmarzyk PT, Lewis CE, Owen N, Perry CK, Siddique J, Yong CM (2016). Sedentary behavior and cardiovascular morbidity and mortality: a science advisory from the American heart association. Circulation.

[44] Quinn TD, Yorio PL, Smith PM, Seo Y, Whitfield GP, Barone Gibbs B (2021). Occupational physical activity and cardiovascular disease in the United States. Occup Environ Med.

[45] Bonekamp NE, Visseren FLJ, Ruigrok Y, Cramer MJM, de Borst GJ, May AM, Koopal C (2023). Leisure-time and occupational physical activity and health outcomes in cardiovascular disease. Heart.

[46] Holtermann A, Mortensen OS, Burr H, Søgaard K, Gyntelberg F, Suadicani P (2009). The interplay between physical activity at work and during leisure time–risk of ischemic heart disease and all-cause mortality in middle-aged caucasian men. Scand J Work Environ Health.

[47] Holtermann A, Krause N, van der Beek AJ, Straker L (2018). The physical activity paradox: six reasons why occupational physical activity (OPA) does not confer the cardiovascular health benefits that leisure time physical activity does. Br J Sports Med.

[48] Kulinski JP, Kozlitina J, Berry JD, de Lemos JA, Khera A (2016). Association between sedentary time and coronary artery calcium. JACC Cardiovasc Imaging.

[49] Nam MW, Lee Y, Lee W (2022). The association between prolonged sedentary time and coronary artery calcification in young healthy men in Korea: a cohort study. Sci Rep.

[50] Lavie CJ, Ozemek C, Carbone S, Katzmarzyk PT, Blair SN (2019). Sedentary behavior, exercise, and cardiovascular health. Circ Res.

[51] Cristi-Montero C, Steell L, Petermann F, Garrido-Méndez A, Díaz-Martínez X, Salas-Bravo C, Ramirez-Campillo R, Alvarez C, Rodriguez F, Aguilar-Farias N (2018). Joint effect of physical activity and sedentary behaviour on cardiovascular risk factors in chilean adults. J Public Health (Oxf).

[52] Papadopoulou SK, Papadimitriou K, Voulgaridou G, Georgaki E, Tsotidou E, Zantidou O, Papandreou D (2021). Exercise and nutrition impact on osteoporosis and sarcopenia-the incidence of osteosarcopenia: a narrative review. Nutrients.

[53] Wu M, Liu Y, Zhong C, Xu B, Kang L (2021). Osteoporosis was associated with severe abdominal aortic calcification based on a cross-sectional study. Arch Osteoporos.

[54] Gielen S, Schuler G, Adams V (2010). Cardiovascular effects of exercise training: molecular mechanisms. Circulation.

[55] Kingwell BA, Arnold PJ, Jennings GL, Dart AM (1997). Spontaneous running increases aortic compliance in Wistar-Kyoto rats. Cardiovasc Res.

[56] Matsuda M, Nosaka T, Sato M, Ohshima N (1993). Effects of physical exercise on the elasticity and elastic components of the rat aorta. Eur J Appl Physiol Occup Physiol.

[57] Li YS, Haga JH, Chien S (2005). Molecular basis of the effects of shear stress on vascular endothelial cells. J Biomech.

[58] Kinlay S, Creager MA, Fukumoto M, Hikita H, Fang JC, Selwyn AP, Ganz P (2001). Endothelium-derived nitric oxide regulates arterial elasticity in human arteries in vivo. Hypertension.

[59] Adams V, Linke A, Kränkel N, Erbs S, Gielen S, Möbius-Winkler S, Gummert JF, Mohr FW, Schuler G, Hambrecht R (2005). Impact of regular physical activity on the NAD(P)H oxidase and angiotensin receptor system in patients with coronary artery disease. Circulation.

[60] Roque FR, Briones AM, García-Redondo AB, Galán M, Martínez-Revelles S, Avendaño MS, Cachofeiro V, Fernandes T, Vassallo DV, Oliveira EM, Salaices M (2013). Aerobic exercise reduces oxidative stress and improves vascular changes of small mesenteric and coronary arteries in hypertension. Br J Pharmacol.

[61] Hamer M, Sabia S, Batty GD, Shipley MJ, Tabák AG, Singh-Manoux A, Kivimaki M (2012). Physical activity and inflammatory markers over 10 years: follow-up in men and women from the Whitehall II cohort study. Circulation.

[62] Shao JS, Cheng SL, Pingsterhaus JM, Charlton-Kachigian N, Loewy AP, Towler DA (2005). Msx2 promotes cardiovascular calcification by activating paracrine wnt signals. J Clin Invest.

[63] Shanahan CM (2007). Inflammation ushers in calcification: a cycle of damage and protection?. Circulation.

