# Supplementary material for: The association of moderate-to-vigorous physical activity and sedentary behaviour with abdominal aortic calcification
Source: J Transl Med. 2023 Oct 9;21:705. doi: 10.1186/s12967-023-04566-w (PMC10563258; doi:10.1186/s12967-023-04566-w)
Supplement: Supplementary file 1 — Additional file 1: Figure S1. Flowchart of the sample selection from NHANES 2013–2014. Missing covariate information (n = 275), including variables such as lower poverty–income ratio (PIR) (N = 250), body mass index (N = 20), education level (N = 4), marital status (N = 3), and smoking status (N = 2). NHANES National Health and Nutrition Examination Survey; AAC abdominal aortic calcification; MVPA moderate-to-vigorous physical activity; SB sedentary behavior. Table S1. Baseline characteristics of the included and excluded populations. [file 12967_2023_4566_MOESM1_ESM.docx]

**Additional file**

Figure S1. Flowchart of the sample selection from NHANES 2013–2014. Missing covariate information (n = 275), including variables such as lower poverty–income ratio (PIR) (N = 250), body mass index (N = 20), education level (N = 4), marital status (N = 3), and smoking status (N = 2). Abbreviation: NHANES, National Health and Nutrition Examination Survey; AAC, abdominal aortic calcification; MVPA, moderate-to-vigorous physical activity; SB, sedentary behavior


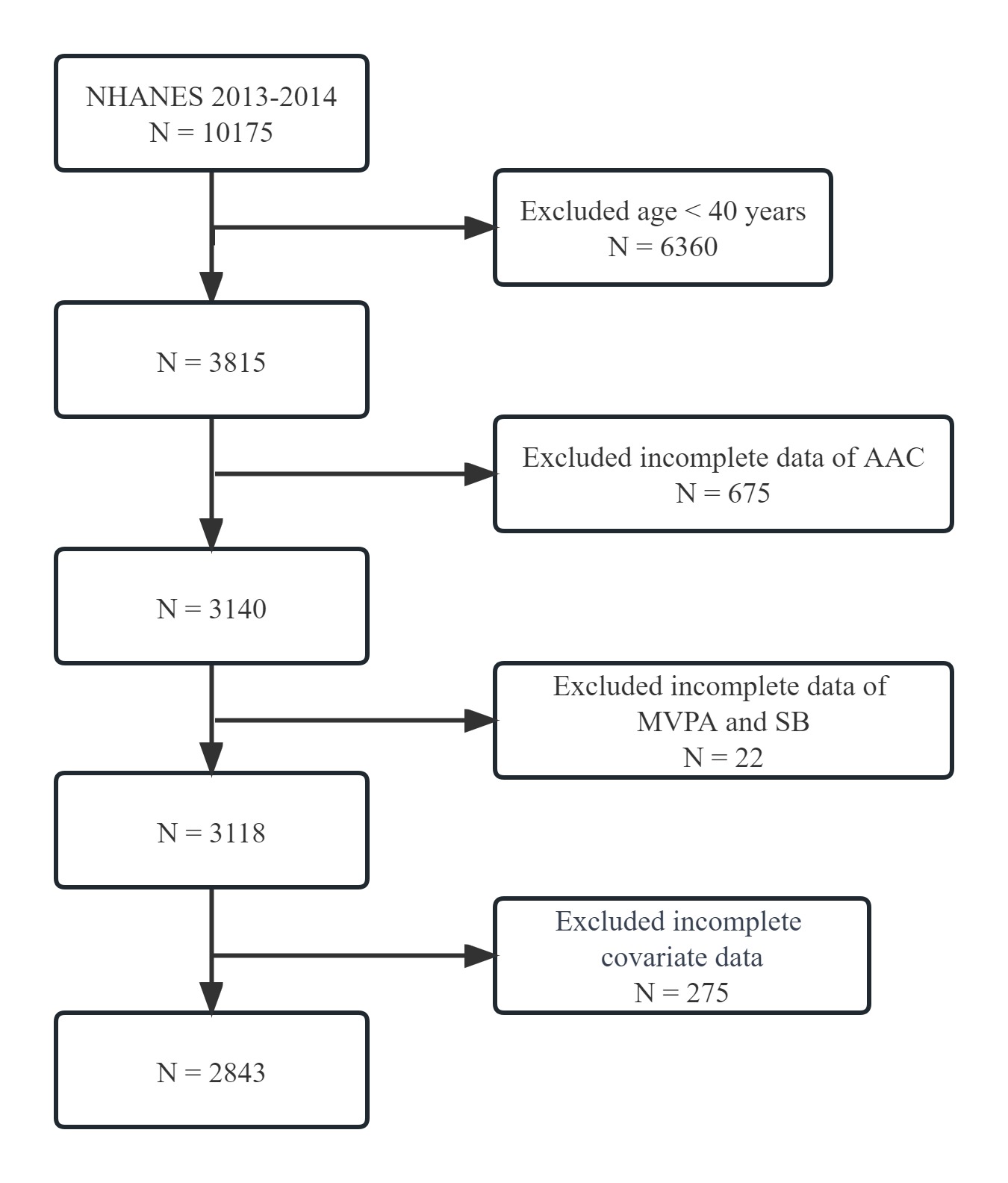


Table S1. Baseline characteristics of the included and excluded populations.

| Characteristic | All (n = 3815) ^a^ | Include (n = 2843) ^a^ | Exclude (n = 972) | *p*-Value |
| --- | --- | --- | --- | --- |
| Age (years) | 59.19 (12.22) | 61.08 (12.68) | 58.54 (11.99) | <0.001*** |
| Sex (%) |  |  |  | 0.024* |
| Male | 1807 (47.37%) | 430 (44.24%) | 1377 (48.43%) |  |
| Female | 2008 (52.63%) | 542 (55.76%) | 1466 (51.57%) |  |
| Race (%) |  |  |  | 0.049* |
| Mexican American | 494 (12.95%) | 147 (15.12%) | 347 (12.21%) |  |
| Other Hispanic | 337 (8.83%) | 81 (8.33%) | 256 (9.00%) |  |
| Non-Hispanic White | 1687 (44.22%) | 401 (41.26%) | 1286 (45.23%) |  |
| Non-Hispanic Black | 782 (20.50%) | 215 (22.12%) | 567 (19.94%) |  |
| Non-Hispanic Asian | 433 (11.35%) | 103 (10.60%) | 330 (11.61%) |  |
| Other Race | 82 (2.15%) | 25 (2.57%) | 57 (2.00%) |  |
| BMI (kg/m2) | 29.33 (6.89) | 32.35 (9.56) | 28.48 (5.64) | <0.001*** |
| Marital status (%) |  |  |  | 0.001** |
| Married/Living with partner | 2355 (61.78%) | 556 (57.38%) | 1799 (63.28%) |  |
| Single | 1457 (38.22%) | 413 (42.62%) | 1044 (36.72%) |  |
| Education level (%) |  |  |  | <0.001*** |
| High school degree/equivalency or less | 1779 (46.68%) | 521 (53.82%) | 1258 (44.25%) |  |
| Some college or associates degree | 1067 (28.00%) | 245 (25.31%) | 822 (28.91%) |  |
| College Graduate or above | 965 (25.32%) | 202 (20.87%) | 763 (26.84%) |  |
| PIR | 2.61 (1.64) | 2.25 (1.57) | 2.69 (1.65) | <0.001*** |

^a^: Missing values for BMI (n = 167), marital status (n = 3), education level (n = 4), PIR (n = 313).

Data is presented as proportions for categorical variables and as means (standard deviations) for continuous variables.

Analysis conducted: Wilcoxon rank sum test and chi-square test.

Abbreviation: BMI, body mass index; PIR, poverty income ratio.

**p* < .05; ***p* < .01; ****p* < .001.
